# Viral and Bacterial Etiology of Common Respiratory Infections in Children in Sub-Saharan Africa: A Systematic Review

**DOI:** 10.3390/children12091212

**Published:** 2025-09-10

**Authors:** Jordy Exaucé Demboux Lyelet, Pembe Issamou Mayengue, Félix Koukouikila-Koussounda, Eric M. Leroy, Pierre Becquart, Fabien Roch Niama

**Affiliations:** 1 Faculty of Sciences and Technics, Marien Ngouabi University, Brazzaville BP 69, Congo; jordydemboux@gmail.com (J.E.D.L.); felixkoukouikila@gmail.com (F.K.-K.); fabien.niama@gmail.com (F.R.N.); 2 National Public Health Laboratory, Brazzaville BP 20, Congo; 3 National Institute for Research in Engineering Science, Innovation and Technology (INRSIIT), Brazzaville Scientific City, Brazzaville BP 181, Congo; 4 UMR TransVIHMI Montpellier University-IRD-Inserm, Montpellier University, 34394 Montpellier, France; eric.leroy@ird.fr; 5 Infectious Diseases and Vectors: Ecology, Genetic, Evolution and Control (MIVEGEC), Institute for Research for Development (IRD), Montpellier University, CNRS, 34394 Montpellier, France; pierre.becquart@ird.fr

**Keywords:** respiratory infection, etiology, viruses, bacteria, sub-Saharan Africa

## Abstract

**Background/Objectives**: Respiratory infections are a major global public health problem, with potentially serious consequences. Indeed, they remain one of the main causes of morbidity and mortality in children under 5 in developing countries. Etiological information on respiratory infections is crucial for prevention and case management strategies. This review describes the etiology of respiratory infections reported in studies conducted in sub-Saharan African countries. **Methods**: PubMed, HINARI and Google Scholar search engines were used for bibliographic research, and only data from sub-Saharan Africa were considered. Articles published between 2010 and 2023, in English or French, were included in this review. **Results**: After a thorough search, 2175 documents were identified. Critical review and removal of duplicates identified 347 full-text studies, which underwent rigorous evaluation. A total of 50 articles were retained, with studies conducted in 24 sub-Saharan African countries, most of them in Cameroon (12%). Thirty-three (66%) were cross-sectional studies, and thirty-seven (74%) were hospital-based surveys. Respiratory syncytial virus was most frequently identified (0.6% to 59%), followed by rhinovirus (7.5% to 73%). The most frequent bacteria were *Streptococcus pneumoniae* (1–96%) and *Haemophilus influenzae* (2.5–54%). **Conclusions**: This study suggests that acute respiratory infections in sub-Saharan Africa, mainly in children, are primarily caused by viruses and a few bacteria.

## 1. Introduction

Respiratory viral infections are increasingly recognized as major contributors to hospitalization and mortality in all age groups worldwide, with a serious form of illness particularly in infants and immunocompromised individuals [1,2]. Annually, lower respiratory tract infections (LRTIs) cause approximately four million deaths worldwide and impart annual global inpatient and outpatient costs of approximately EUR 5 billion [3,4].

Most epidemiological knowledge is based on data from developed countries. In contrast, the burden of acute respiratory infections (ARI) is particularly heavy among children in developing countries, with high rates of hospital admissions and mortality [5,6]. Indeed, it is estimated that about 126 to 156 million cases of acute lower respiratory tract infections (ALRTI) such as pneumonia and bronchiolitis occur in children worldwide each year, causing around 1.4 million deaths, over 95% of which occur in Africa and Southeast Asia [7].

Upper respiratory tract infections are commonly caused by viruses or bacteria. Respiratory viruses are more often responsible for upper tract ARIs than bacteria in children under 5 years of age [6]. Common symptoms include nasal congestion, cough, sore throat, and fever. However, bacteria are less identified because of low sensitivity of bacterial culture, particularly in patients with community-acquired pneumonia [8]. Respiratory viruses such as respiratory syncytial virus, Influenza viruses (A and B), parainfluenza viruses, human adenovirus, human coronaviruses OC43 and 229E, rhinovirus and metapneumovirus are currently recognized as common etiologies of ARI in young children in developed countries [5].

Recent use of molecular diagnostic techniques has identified other respiratory viruses associated with ARI, including human metapneumovirus, human Bocavirus, human coronavirus NL63, and human coronavirus HKU1 [6]. In addition, human rhinovirus is implicated in the majority of cold cases and often induces lower respiratory tract infections [6]. A better understanding of the range of pathogens responsible for ARI is therefore essential for clinical case management and the design of preventive strategies aimed at reducing childhood morbidity and mortality.

Lower respiratory tract infection (LRTI) is common in the elderly, children under five years of age and people who are immunocompromised or suffering from co-morbidity [9]. People with symptoms suggestive of LRTIs can contract tuberculosis (TB) and/or other bacterial and viral infections [10]. Over the years, the most severe cases of pneumonia have been associated with *Mycobacterium tuberculosis*, with little information on other relevant bacterial pathogens [11]. Some common pathogens causing LRTIs other than *Mycobacterium tuberculosis* include: *Streptococcus pneumoniae*, *Haemophilus influenzae*, *Klebsiella pneumoniae* and *Staphylococcus aureus* [11].

The viral and bacterial etiologies of ARIs have been well documented in Northern Hemisphere countries [12,13]. However, few studies are available in Africa [14]. Thus, the present study aims to summarize the literature related to the etiology of respiratory infections in sub-Saharan African countries and to identify information gaps to improve essential knowledge on the subject.

We focused our research on sub-Saharan Africa, as epidemiological, socioeconomic and vaccine policy factors in North Africa would probably be very different [15]. Indeed, the distinction between North Africa and sub-Saharan Africa is climatically and ecologically significant because of the natural barrier created by the Sahara Desert, the world’s largest desert with a harsh, hot climate [16].

## 2. Methods

The Preferred Reporting Items for Systematic Reviews and Meta-Analyses (PRISMA) was followed for our review [17]. This review was registered on the Open Science Framework; Registration DOI: https://doi.org/10.17605/OSF.IO/EXRCS (https//osf.io/nsd5m/, accessed on 29 July 2024).

### 2.1. Search Strategy

This review considers data from documents published online (articles, reports, etc.) that reported information on both viral and bacterial etiology of ARIs in sub-Saharan Africa, by searching the online bibliographic databases PubMed, HINARI and Google Scholar using the following key terms: “Acute respiratory infections”, “Upper respiratory infections”, “Lower respiratory infections”, “Viruses”, “Bacteria”, “Respiratory syndrome”, “Influenza syndrome”, “sub-Saharan Africa”, “Prevalence/Proportion”, and “etiology”. The reference list of selected articles was used as a lead for identifying further studies. The Boolean operators “AND” and “OR” were used to combine two or more terms. The search was limited to studies published in English or French, involving patients of any age in sub-Saharan Africa, in which pathogens were identified using immunofluorescence assays (IFA), Polymerase Chain Reactions (PCR), viral cultures, bacterial cultures, or a combination of these methods.

We also adopted the Preferred Reporting Items for Systematic Reviews and Meta-Analyses (PRISMA) checklist [17]. Two research questions guided this review: (1) What is the etiology of respiratory infections in sub-Saharan Africa, viral and/or bacterial? (2) What are the positive proportions of these pathogens in each study?

### 2.2. Study Selection

This review compiles studies focused on ARIs caused by viruses and/or bacteria. We only considered data from sub-Saharan Africa reported in papers published between 2010 and 2023, in English or French.

### 2.3. Inclusion Criteria

Studies included were cohort, analytical, prospective, retrospective, and cross-sectional investigations reporting the proportion of respiratory viruses and bacteria in hospital and/or community settings. In the case of repeated studies, where the same population was recruited and examined over the same period, only the most recent or most complete study was included.

### 2.4. Exclusion Criteria

**Figure 1:**
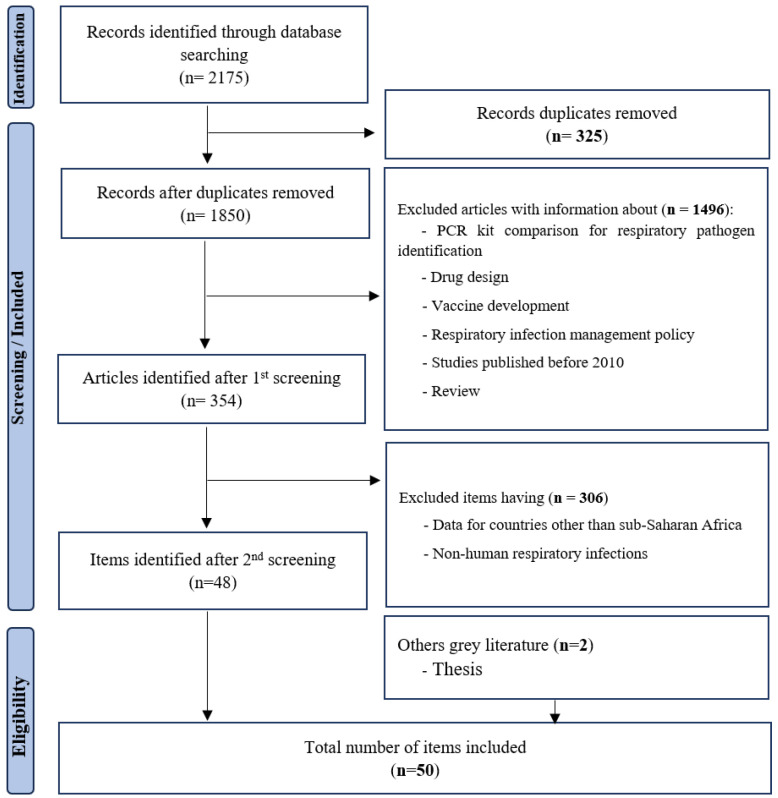
Summary of search strategy (PRISMA flow diagram).

There were no age or gender restrictions (Figure 1). Exclusion criteria were mainly: (i) respiratory infections of non-human origin; (ii) comparison of PCR kits for identification of respiratory pathogens; and (iii) studies on respiratory infection management policy. Endnote software version X9 Bld 12062, was used to remove duplicates and manage records during the screening process.

### 2.5. Data Extraction

Full versions of selected articles were downloaded and reviewed by two study authors. Data were extracted using a predefined form with the following information: (i) references; (ii) sample collection period; (iii) year of publication; (iv) study country; (v) age range; (vi) study objective; (vii) zone/sample size; (viii) study framework; (ix) type of sampling; (x) diagnostic methods; (xi) proportion of pathogens; and (xii) type of study.

### 2.6. Data Summary

We synthesized the data by summarizing the main findings of each study. Given the variety of study types included in the review, ranging from simple descriptive to analytical studies, we have considered a synthesis more appropriate rather than a formal meta-analysis. Tables were created to list all the pathogens found in each study, together with relevant study information as mentioned above on data extraction.

## 3. Results

### 3.1. Literature Review

The published articles included in this review were those from studies with samples collected from 2006 to 2022. To filter articles for this review, we initially identified a total of 2168 articles from PubMed, HINARI and Google Scholar that fit with our initial search strategy. Of these, 50 studies were included and 2125 were excluded, after screening each article (Figure 1).

Of the 50 studies included, 9 focused on viral and bacterial strains responsible for pneumonia in children and the elderly [18,19,20,21,22], and 41 focused on the surveillance and epidemiology of viral or bacterial strains responsible for respiratory infections. Among them, 36 studies were carried out in outpatients, whereas 14 studies were from hospitalized patients (Table 1 and Table 2). All viruses and bacteria found in hospitalized patients were also identified in outpatients.

Many of these studies were carried out among children under 5 years of age. Articles excluded were related to comparisons of amplification kits, respiratory infection management policy, data from countries other than Africa, and those concerning non-human respiratory infections.

### 3.2. Features of Included Studies

**Figure 2:**
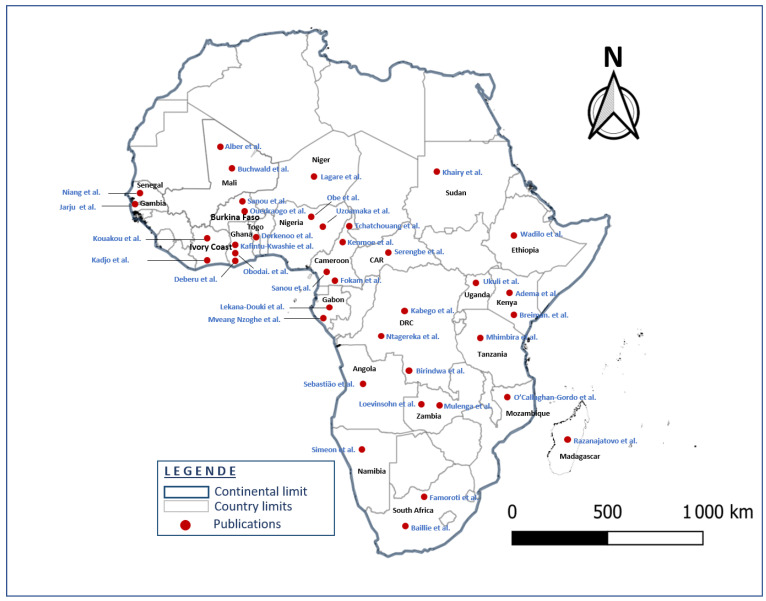
Geographical identification of the 50 included studies associated with the 24 countries (map generating with QGIS 3.16.0).

The 50 studies involved a total of 81,621 patients. Sample size ranged from 91 to 14,119 ARI patients per study. The included studies were conducted in 24 sub-Saharan African countries (Figure 2).

A total of 6 published studies were conducted in Cameroon; 4 in Ghana, Gabon and Burkina Faso; 3 each in the Democratic Republic of the Congo (DRC), Nigeria, Zambia and Kenya; 2 studies each in South Africa, Ivory Coast, Niger and Mali, respectively; and one study in each of the following countries: Senegal, Tanzania, Ethiopia, Central African Republic (CAR), Angola, Mozambique, Uganda, Togo, Namibia, Madagascar, Gambia and Sudan.

**Figure 3:**
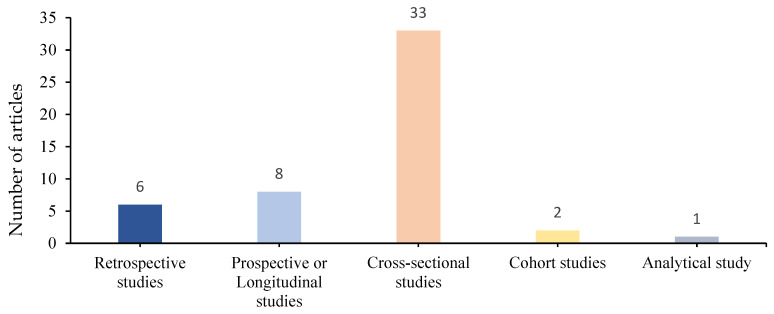
Distribution of different types of studies.

We identified 33 (66%) cross-sectional (descriptive and case–control) studies, 8 (16%) prospective or longitudinal studies, 6 (12%) retrospective studies, 2 (4%) cohort studies and 1 (2%) analytical study (Figure 3).

Considering settings in which these published studies were focused, there were 37 (74%) hospital-based studies, 11 (22%) community-based studies, and 2 (4%) were not indicated. The study setting was urban in 42 (84%) studies, and mixed (rural, semi-rural and pre-urban) in 8 (16%) studies.

Pathogens were identified in a variety of respiratory samples, including nasal swabs, oropharyngeal swabs, nasopharyngeal aspirates, induced sputum, tracheal aspirates, bronchoalveolar lavage swabs, urine, blood and pulmonary aspirates.

Respiratory viruses were detected using immunofluorescence tests, multiplex/simplex RT-PCR, conventional PCR, blood culture and viral cultures (Table 1). RT-PCR was the most frequently used diagnostic method. For the detection of individual bacteria, only bacterial cultures and PCR were performed.

### 3.3. Etiology of Pathogens Detected

All the respiratory pathogens identified in these studies were viral and bacterial (Table 1 and Table 2). Among 50 studies reviewed, human respiratory syncytial virus was the most frequently identified, with a proportion ranging from 0.6 to 59%, followed by human rhinovirus (7.5–73%), Influenza A/B virus (0.9–69.1%), human adenovirus (0.9–30.8%), human parainfluenza virus 1–4 (2–24%), enterovirus (2.9–25.5%), human coronaviruses (1.4–13.9%), human metapneumovirus (1–23.3%), SARS-CoV-2 (0.4–44%) and human bocavirus (1.4–16.2%) (Table 3).

Among the bacteria detected (Table 1 and Table 2), the most prevalent were *Streptococcus pneumoniae* (1–96%), followed by *Haemophilus influenzae* type b (2.5–54%), and *Klebsiella pneumoniae* (1.4–49.9%). Other bacterial species, notably *Staphylococcus aureus* (1.7–12.2%), *Pseudomonas aeruginosa* (1.4–37.5%), *Mycobacterium tuberculosis* (6.5%), *Salmonella typhi* (1.6%) and other very rarely identified bacteria, such as: *M. catarrhalis* (46.2%), *B. Pertussis* (0.1%), and *Enterobacter* sp. (22.2%) (Table 3).

## 4. Discussion

Respiratory infections constitute one of the major public health problems with an important socioeconomic impact [3,4]. Recently, SARS-CoV-2 infection, with clinical manifestations similar to those of common respiratory viruses, showed how often a respiratory infection may become pandemic and revealed the fragility of healthcare systems, particularly in sub-Saharan Africa [64]. Thus, knowledge of the etiology of respiratory pathogens is essential for better management of infections.

This review updates known information on respiratory infections of viral and/or bacterial etiology in sub-Saharan Africa over the last twelve years. The overall goal of this systematic review was to inform public health actors and researchers on the etiology of respiratory infections (viral and bacterial) in Africa and to provide information that can support actions to optimize decision-making by health authorities for the control of these infections.

A wide variety of detection techniques were found in this review, including molecular viral detection and bacterial culture, which is the universal and reference method for the characterization of respiratory infection pathogens. However, other tools such as neutrophil to lymphocyte ratio (NLR) have been recently successfully tested for an early differential diagnosis of pneumonia’s etiology in children in Egypt and Italy [65,66] and could be used in sub-Saharan Africa, mainly where financial resources are limited. As demonstrated in adults [67,68], NLR is a relevant diagnostic tool that reflects the imbalance between innate and adaptive immunity, and its recent pediatric application confirms its potential in the early identification of respiratory infectious causes. The results highlight a predominance of human respiratory syncytial virus and a strong association between human rhinovirus and Influenza A/B virus in children aged below 5 years, presenting with influenza-like illness. The other most frequently detected viruses were adenovirus and all four types of human Parainfluenza virus. This study also showed respiratory infections of bacterial origin, with the most frequently identified species being *Streptococcus pneumoniae* and *Haemophilus influenzae*, mainly in bacterial culture as well as in sputum and Brancoalveolar lavage (BAL) samples in adults. These findings may not globally reflect the real picture of different pathogens associated with respiratory infections. Thus, comparison with available WHO African Region reports is needed.

Little or no data were found on the etiology of respiratory infections in many sub-Saharan African countries. Of the 48 countries in sub-Saharan Africa (wikipedia.org/wiki/Afique_sub-saharienne, accessed on 28 September 2023), the 50 studies included in this review were carried out in only 24 countries, the majority of which were in Central and West Africa (Figure 2). No published studies were carried out in the Republic of the Congo, although it borders two (Cameroon and Democratic Republic of the Congo) of the five countries where the number of deaths from childhood pneumonia was the highest [69]. This lack of data could probably be due to the poor implementation of respiratory infection surveillance activities.

The pattern of predominance of human respiratory syncytial virus in this study is consistent with that reported by several previous systematic reviews [6,70,71,72]. Regardless of various factors, including screening test, type of sample tested, age of children, type of education, and severity of infection, most studies indicated that human respiratory syncytial virus is the predominant causative agent of cases of respiratory diseases such as bronchiolitis, asthma, and wheezing with an incidence of 50–80% [73]. Rhinovirus and Influenza A/B, the second most common viruses observed, have long been considered a cause of benign respiratory tract infections such as the common cold [14].

We found five studies that presented cases of viral and bacterial co-infections at rates of around 14% in our review [14,19,20,21,22]. Although *Streptococcus pneumoniae* is known to be more prevalent in superinfection in some respiratory syndromes, such as Influenza [74,75], *Haemophilus influenzae* and *Klebsiella* spp. were also identified mostly in co-infection. This observation correlates with the review by Lansbury et al., who also showed that *Klebsiella pneumoniae* and *Haemophilus influenzae* were among the most frequent co-infecting bacterial pathogens even in patients with COVID-19 [76,77]. *Staphylococcus aureus* was one of the least present, as expected [18]. Irrespective of testing issues, co-infection with other respiratory pathogens has important implications for diagnosis and prognosis.

Seasonality and study duration could clearly also lead to variability in the proportion of viruses/bacteria responsible for respiratory infections.

## 5. Conclusions

This review shows that a number of viruses are associated with ARIs in children and adults in sub-Saharan Africa. The WHO’s global strategy for the control of ARI in children under 5 years of age must rigorously consider the importance of both viral and bacterial cases. Moreover, the results highlight the lack of data for several sub-Saharan African countries. Further high-quality studies are needed to determine the role of viruses and bacteria in ARI. In this vein, an approach combining the scientific studies and institutional reports should be considered to provide adequate epidemiological and etiological information.

## 6. Study Limits

This study has several limitations: (1) Only publications in English or French were taken into account, excluding data published in Portuguese, which is the official language of five African countries (Angola, Cape Verde, Guinea-Bissau, Mozambique and São Tomé and Principe), and in Spanish, the official language of Equatorial Guinea. (2) The unpublished literature also constitutes an information bias in this systematic review. Finally, we did not assess the statistical quality of the studies by meta-analysis but included all articles that met the inclusion criteria. (3) Although the WHO reports are an essential source for global surveillance of respiratory infections, producing annual reports on regional or continental global health statistics [78], we have chosen to focus on more detailed scientific sources specific to our study context, with more detailed information on etiological specificity.

## Figures and Tables

**Table 1:** Summary of published studies carried out in outpatients.

References	Collection Period	Year of Publication	Study Country	Age Range	Study Objective	Zone/Sample Size	Study Framework	Type of Sampling	Diagnostic Methods	Proportion of Pathogens	Type of Study
Lekana -Douki et al. [23]	2009–2011	2013	Gabon	No limit	To report the results of a large surveillance study for pH1N1 in Gabon during a 2-year period, July 2009–June 2011	Urban/966	Health care centers The regional hospitals	Nasal	RT-PCR	Flu A (61%); Flu B (39%)	Cross-sectional/Prospective
Lekana- Douki et al. [24]	2010–2011	2014	Gabon	No limit	To determine the prevalence, etiology and seasonality of viral respiratory tract infections	(Urban)/1041	Health care centers The regional hospitals	Nasopharyngeal	One-step multiplex real-time RT-PCR	HAdV (17.5%), HPIV 1–4 (16.8%), EV (14.7%), HRSV (13.5%), and Flu A (11.9%)	Cross-sectional
Ouédraogo et al. [5]	2010–2011	2014	Burkina Faso	<3 years	To identify the respiratory viruses, present in children admitted to or consulting at the pediatric hospital in Ouagadougou	(Urban)/209	Charles de Gaulle pediatric hospital	Nasopharyngeal	One-step multiplex real-time RT-PCR	HRV (59.1%); EV (25.5%); HRSV (16.1%); HMPV (9.4%)	Prospective
Breiman et al. [25]	2007–2011	2015	Kenya	<5 years	To analyze data from our population-based infectious disease surveillance (PBIDS) site in Kibera, an urban slum in Nairobi	2592	Community (Households)	Blood Naso/Oro-pharyngeal	Hemoculture RT-qPCR	HRV/EV (42%); HRSV (25%); HAdV (20%); HMPV (13.7%), Flu A (10.8%); *Salmonella typhi* (1.6%); *Streptococcus pneumoniae* (1%); *Staphylococcus aureus* (1.7%)	Cross-sectional
Serenbe et al. [26]	2013	2015	Central African Republic	<5 years	To determine the contribution of viruses to respiratory infections in children under five.	361	Referral hospital Outlying pediatric centers	Nasopharyngeal	RT-qPCR Multiplex RT-PCR	HRV (47.5%); FluA/B (26.6%); HPIV-3(9.3%); HRSV (5.8%); EV (4.3%); HAdV (2.9%); HBoV (1.4%); HCoV (1.4%)	Cross-sectional
Kenmoe et al. [27]	2011–2013	2016	Cameroon	≤15	To investigate the viral etiology and seasonality of SARI in hospitalized children in Yaoundé, Cameroon	(Urban)/347	Hospital (pediatric service)	Nasopharyngeal	Multiplex PCR RT-PCR	HRSV (13.2%), HAdV (27.3%), HboV (10.6%), Flu A/B (9.8%); HPIV (6.6%); HCoV (5.7%); HMPV (2.3%); HRV/EV (11.5%)	Prospective
Uzoamaka et al. [28]	2014–2016	2017	Nigeria	No limit	To determine the current trends of bacterial etiology of LRTIs among the patients that attended the University of Nigeria Teaching Hospital (UNTH), and their antimicrobial susceptibility profile with special interest in pan drug resistance	(Peri-urban) /954	Hospital	Expectoration	Culture (MacConkey agar, blood agar, chocolate agar)	*Klebsiella pneumonia* (49.9%); *Klebsiella* spp./*Pseudomonas aeruginosa*, (1.4%)	Cross-sectional
Niang et al. [29]	2012–2015	2017	Senegal	No limit	To investigate the epidemiologic and viral molecular features of HAdVs circulating in Senegal after 4 consecutive years of sentinel surveillance of influenza like illness cases	(Urban) /6381	Hospital	Naso/Oro-pharyngeal	Two-step multiplex real-time RT-PCR	HAdV (30.8%); FluA/B (53.1%); HRV (30%); Ev (18.5%); HRSV (13.5%)	Cross-sectional/ Prospective
Famoroti et al. [30]	2011–2015	2018	South Africa	0–5 years	To determine the most common viral pathogens associated with ARTI among children between 0 and 5 years of age in KwaZulu-Natal	(Urban) /2172	Hospital (pediatric service)	Expectoration /Nasopharyngeal	Multiplex PCR	HRSV (32.1%), HAdV (21.8%), HRV (15.4%), FluA swl (5.1%)	Retrospective
Kadjo et al. [31]	2013	2018	Ivory Coast	<5 years	To describe the epidemiological, clinical, and virological pattern of ARI that tested negative for influenza virus, in children under five years old	(Urban) /1340	Hospital (pediatric service)	Nasopharyngeal	RT-PCR	HRV (31.92%), HRSV (24.4%), HPIV (20.5%), HCoV 229E (12.05%)	Cross-sectional
Sanou et al. [32]	2014–2015	2018	Burkina Faso	<5 years	To assess the prevalence and seasonal occurrence of influenza viruses in children with ILI and severe acute respiratory infection (SARI)	(Urban) /924	Healthcare centers	Nasopharyngeal	RT-PCR	Flu A/B (15.1%), A(H3N2) (69.1%) A(H1N1) pdm09 (30.9%)	Cross-sectional
Obodai et al. [33]	2006, 2013–2014	2018	Ghana	<5 years	To assess HRSV diagnostics and/or surveillance in affected age groups in the future and to the molecular understanding of the HRSV circulation in Ghana, Africa	(Urban) /552	Hospital (pediatric service)	Nasopharyngeal	RT-PCR	HRSV (23%)	Cross-sectional
Lekana-Douki et al. [34]	2018	2018	Gabon	<5 years	To evaluate the prevalence and the HBoV genotype in children under 5 years old with ILI or diarrhea in Gabon	(Urban) /810	Health centers	Nasopharyngeal	RT-PCR	HBoV (4.4%)	Retrospective
Kabego et al. [35]	2016	2018	Democratic Republic of the Congo	<5 years	To determine the prevalence of human respiratory syncytial virus (HRSV) acute respiratory infection (ARI) in children under the age of 5 years at the Provincial General Hospital of Bukavu (PGHB), and to analyze factors associated with the risk of ARI being diagnosed as lower respiratory tract infection (LRTI)	(Urban) /146	General Hospital	Nasopharyngeal	Multiplex RT-PCR	HRSV (21.2%); HRV (16.4%); HPIV-3 (16.6%) and HAdV (4.79%).	Cross-sectional, analytical/Prospective
Mhimbira et al. [21]	2013–2015	2018	Tanzania	No limit	To describe the prevalence of respiratory pathogens in TB patients and household contact controls, and the clinical significance of respiratory pathogens in TB patients	(Urban) /972	Community (Households)	Nasopharyngeal	Multiplex RT-PCR	HRV (9.3%); Influenza A (3.1%); HRSV A (1.9%); *H. influenzae* (26.1%); *S. pneumoniae* (21.5%)	Prospective cohort
Kenmoe et al. [36]	2011–2014	2018	Cameroon	<15 years	To document the different types of HAdV circulating in Cameroon in children with acute respiratory infections	(Urban) /811	Hospital (pediatric service)	Nasopharyngeal	RT-PCR	HAdV (27.12%)	Cross-sectional
Razanajatovo et al. [22]	2010–2013	2018	Madagascar	No limit	To identify etiologies and describe clinical features of SARI-associated hospitalization in Madagascar	(Urban) /876	Hospital- based	Nasopharyngeal, Expectorations Blood	Multiplex RT-PCR Cytobacteriologic testing	HRSV (37.7%); FluA (18.4%); HRV (13.5%; HAdV (8.3%); *S. Pneumoniae* (50.3%); *H. Influenzae b* (21.4%); *Klebsiella* (4.6%)	Prospective
Tchatchouang et al. [18]	2019	2019	Cameroon	No limit	To identify the respiratory bacteria of patients presenting with symptoms and clinical signs of LRTI at a referral center for respiratory diseases in Yaoundé, Cameroon	(Urban) /141	Hospital (Pneumology department)	Branco-alveolar lavage (BAV)	Bacterial Culture	*S. pneumoniae*/*H. infuenzae* (14.2%); *K. pneumoniae* (9.2%); *S. aureus*, (7.1%)	Prospective
Adema et al. [37]	2017–2018	2020	Kenya	<20 years	To advance understanding of the nature of spread of respiratory viruses	(Urban) /781	Community (School)	Nasopharyngeal	Multiplex RT-PCR	HRV (16.7%); HPIV (2.7%); HCoV (229E, NL63, OC43) (2.0%); HAdV (0.9%); HRSV (0.6%)	Longitudinal/ Cohort
Jarju et al. [3]	2018–2019	2020	Gambia	<5 years	To determine the viral etiology, seasonality, clinical features and associated AMU	(Urban) /805	Hospital (Medical Research Council Unit)	Nasopharyngeal	Multiplex RT-PCR	HRV (36.7%); Flu A (7.0%); Flu B (1.4%); HRSV (14.7%); HMPV (7.2%); HPIV (13.2%); HCoV (229E, OC43 or NL63) (7.8%)	Prospective
Buchwald et al. [38]	2011–2013	2020	Mali	<2 years	To provide the first estimates of RSV incidence in Mali	(Urban) /1333	Community (Households)	Naso/Oro-pharyngeal	RT-PCR	HRSV (37%)	Cohort
Obe et al. [39]	2021	2021	Nigeria	<5 years	To determine molecular prevalence of RSV among under five children admitted with ALRTTIs in a tertiary hospital and identify the risk factors associated with the acquisition of RSV-ALRTIs	(Urban) /200	Hospital (pediatric service)	Nasopharyngeal	RT-PCR	HRSV (22.5%)	Cross-sectional
Deberu et al. [11]	2018–2019	2021	–Ghana	No limit	To identify the presence of pathogens in sputum of suspected tuberculosis cases and their antimicrobial resistance patterns	(Urban) /264	Public Health Laboratory	Expectoration	Culture (MacConkey agar, blood agar, chocolate agar)	*Klebsiella* spp. (28%); *M. tuberculosis* (6.5%); *Pseudomonas* spp. (15.2%)	Retrospective
Kouakou et al. [40]	2021	2021	Ivory Coast	≤5 years	To provide general knowledge on the epidemiology of the virus, its seasonality and the signs associated with RSV in children aged 0 to 5 years in Côte d’Ivoire	(Urban/rural)/5648	Hospitals University (CHU) Regional Hospitals (CHR), General Hospitals (HG) Community Urban Health Unit	Nasopharyngeal	RT-qPCR	HRSV (10%)	Cross-sectional/descriptive
Kenmoe et al. [41]	2011–2014	2021	Cameroon	No limit	To report the occurrence and phylogenetic relatedness of EVs and RVs detected in samples from patients of all ages suffering from ARI in Cameroon based on the sequences of the VP4/VP2 genomic region	(Urban) /974	Health centers	Nasopharyngeal	RT-PCR	HRV/EV (16.4%)	Cross-sectional
Birindwa et al. [20]	2015–2017	2021	Democratic Republic of the Congo	≤5 years	To describe clinical characteristics and risk factors and to determine the occurrence of bacteria and viruses in the nasopharynx of hospitalized children with pneumonia in the Eastern DR Congo	(Urban) /2322	Hospital	Nasopharyngeal	Multiplex RT-PCR assay Culture	*H. influenzae* (54%); *S. pneumoniae* (96%); HRV (73%); EV (17%); HRSV (7%);	Cross-sectional
Ntagereka et al. [42]	2021	2022	Democratic Republic of the Congo	No limit	To investigate the prevalence of SARSCoV-2, influenza A and B, and other acute respiratory viruses among local patients with flu-like symptoms	(Urban) /1352	Hospital, Health Center.	Oro-pharyngeal	RT-PCR	SARS-CoV-2 (13.9%), Flu A (5.6%), Flu B (0.9%)	Cross-sectional
Kafintu- Kwashie et al. [43]	2015–2016	2022	Ghana	<5 years	To investigate and genotype respiratory syncytial virus and human metapneumovirus in children presenting with ALRTI infections at the Princess Marie Louis Children’s Hospital in Accra, Ghana	(Urban) /188	Hospital	Nasopharyngeal	Two-step RT-PCR	HRSV (11.4); HMPV (1.7%);	Cross-sectional
Kolawole et al. [44]	2017	2017	Nigeria	<14 years	To investigate if clinical cases may describe the entire picture of ARI among children in Nigeria	(Urban) /91	Community Hospital	Nasopharyngeal	PCR	HcoV OC43 (13.3%); HcoV 229E/NL63 (12.5%)	Cross-sectional
Ukuli et al. [45]	2008–2016	2023	Uganda	No limit	To identify and characterize new and re-emerging adenoviruses, which is important in the prevention and control of disease outbreaks as it would aid in predicting and preparing for future disease occurrences	2298	Hospital	Nasopharyngeal	PCR	HAdV (9.8%)	Retrospective
Feikin et al. [46]	2007–2010	2012	Kenya	No limit	To report bacterial and viral etiologies of ARI by age group, hospitalization status, HIV infection status and season. We also provide incidence by etiology, adjusted for healthcare seeking and presence of pathogens in asymptomatic controls	(Rural) /3046	Community Hospital	Naso/Oro-pharyngeal Blood Urine	RT-qPCR Culture	HRV/EV (33%); Flu A (22%); *S. pneumoniae* (3%); *Salmonella* sp. (3%)	Cross-sectional
Fokam et al. [47]	2020–2021	2022	Cameroon	No limit	To conduct a study in a large number of individuals tested for the presence of SARS-CoV-2 by PCR during the first epidemiological wave, to shed more light on the epidemiological, virological and clinical characteristics of COVID-19 in Cameroon	Urban/14119	Center for research	Nasopharyngeal	RT-PCR	SARS-CoV-2 (12.7%)	Cross-sectional
Dorkenoo et al. [48]	2020	2022	Togo	No limit	To estimate the prevalence of malaria and COVID-19 in febrile patients in Lomé	Urban/243	Community health center of Legbassito; Hospital of Bè; Centre Hospitalier Universitaire Campus	Nasopharyngeal	GeneXpert	SARS-CoV-2 (7.4%)	Cross-sectional
Alber et al. [49]	2020–2021	2022	Mali	No limit	To estimate the degree of severe acute respiratory syndrome coronavirus-2 (SARS-CoV-2) transmission among health care workers (HCWs) and the general population in a West African setting	Urban/2392	Referral and community health center	Oro-pharyngeal	RT-PCR	SARS-CoV-2 (2.8%)	Cohort
Khairy et al. [50]	2020–2021	2023	Sudan	<19 years	To describe the epidemiology of COVID-19 in children and adolescents in Sudan during 2020–2021	Urban/3150	Not indicated	Nasopharyngeal	PCR	SARS-CoV-2 (44%)	Cross-sectional
Mulenga et al. [51]	2020	2021	Zambia	No limit	To estimate SARS-CoV-2 prevalence in six districts of Zambia in July 2020, using a population-based household survey	Urban/2848	Households	Nasopharyngeal	RT-PCR	SARS-CoV-2 (7.6%)	Cross-sectional

Abbreviations: HRSV: Human Respiratory Syncytial Virus; HRV: Human Rhinovirus; HAdV: Human Adenovirus; Flu A/B: Influenza viruses A/B type; HPIV 1–4: Human Parainfluenza viruses 1–4 types; EV: Enterovirus; hCoV: Human Coronavirus; hMPV: Human Metapnemovirus; hBoV: Human Bocavirus; SARS-CoV-2: Severe acute respiratory syndrome coronavirus 2; HIV: Human Immunodeficiency Virus; *S. pneumoniae*: *Streptococcus pneumoniae*; *S. aureus*: *Staphylococcus aureus*; *H. influenza* b: *Haemophilus influenzae* b type; *K. pneumoniae*: *Klebsiella pneumoniae*; *P. aeruginosa*: *Pseudomonas aeruginosa*; *M. tuberculosis*: *Mycobacterium tuberculosis*; RT-qPCR: quantitative Real Time Polymerase Chain Reaction; RT-PCR: Reverse Transcription Polymerase Chain Reaction.

**Table 2:** Summary of published studies carried out in hospitalized patients.

References	Collection Period	Year of Publication	Study Country	Age Range	Study Objective	Zone/Sample Size	Study Framework	Type of Sampling	Diagnostic Methods	Proportion of Pathogens	Type of Study
Lagare et al. [14]	2010–2012	2015	Niger	<5 years	To document the prevalence of selected viral and bacterial infections among children <5 years of age hospitalized with severe acute respiratory illness (SARI)	(Urban)/160	Hospital	Nasopharyngeal	One-step multiplex RT-PCR	HRSV (35%); HRV (29%); HPIV (24%); *S. pneumoniae* (56%); *H. inflenzae* (12%)	Retrospective
Lagare et al. [19]	2015	2019	Niger	<5 years	To describe viral and bacterial infections among children aged younger than 5 years hospitalized with febrile ARI at two hospitals in Niamey, Niger’s capital city, and the reported clinical procedures.	(Urban)/638	Hospital	Expectoration /Nasopharyngeal	RT-qPCR	HRSV (23.3%), HPIV (12.2%), HRV (9.4%), HAdV (9.4%), Flu A (8.1%)/*S. pneumoniae* (39%), *S. aureus* (12.2%), *H. influenzae B* (2.5%)	Prospective
Wadilo et al. [52]	2019–2022	2023	Ethiopia	<5 years	To estimate the contribution of respiratory viruses to LRTIs among hospitalized children younger than 5 years.	(Urban)/420	Hospital	Naso/Oro-pharyngeal	RT-qPCR	HRSVA/B (30.5%); HRV (18.6%); HBoV (16.2%); HMPV (23.3%); SARS-CoV-2 (0.47%)	Prospective case–control
Baillie et al. [53]	2011–2014	2021	South Africa	≤5 years	To focus on the clinical epidemiology of RV infection, overall and by site, and its interactions with other respiratory pathogens in children 1–59 months of age hospitalized with pneumonia and in community controls.	(Urban)/4232	Hospital	Naso/Oro-pharyngeal	RT-PCR	HRV (21%)	Cross-sectional
Simusika et al. [54]	2011–2012	2015	Zambia	<5 years	To detect upper respiratory pathogens in specimens that were previously laboratory-confirmed influenza negative, to investigate the relative frequency of isolation, seasonality, and clinical diagnosis of various pathogens identified from SARI patients	(Urban)/496	Hospital	Naso/Oro-pharyngeal	Singleplex and multiplex rRT-PCR	HRV (19.2%); HADV (16.8%); HRSV (15.2%); HMPV (4.7%); *S. pneumoniae* (54.8%); *M. catarrhalis* (46.2%); *H. influenzae* (40.7%)	Cross-sectional
Loevinsohn et al. [55]	2018–2019	2021	Zambia	No limit	To describe the diversity of pathogens in the nasopharynx among patients with respiratory infections presenting for care in rural Zambia and the prognostic implications of co-infection.	(Rural)/671	Hospital	Nasopharyngeal	RT-PCR (GeneXpert)	Flu A (12.7%); Flu B (6.4%); HRSV (4.4%); HRV (26.13%); HPIV (2.08%) HMPV (1.01%); HCoV (6.27%); HAdV (2.63%); *Bordetella pertussis* (0.19%)	Cross-sectional
Ouédraogo et al. [56]	2010–2011	2016	Burkina Faso	<5 years	To investigate the prevalence of RSV viral infections in infants suffering from respiratory infections and hospitalized in the city of Ouagadougou, and to evaluate the clinical characteristics associated with the identification of RSV.	(Urban)/209	Hospital	Nasopharyngeal	RT-PCR	HRSV (16.2%)	Cross-sectional
Kenmoe [57]	2011–2014	2017	Cameroon	<15 years	To determine the etiology of ARI in children hospitalized in Yaoundé, Cameroon, and to genetically characterize the HRSV and HMPV strains detected.	(Urban)/822	Hospital	Nasopharyngeal	PCR/RT-PCR	HRSV (9%); HMPV (3.9%); HAdV (28.5%); Flu A/B (21.4%); HRV/EV (15.5%); HBoV (9.4%); HCoV (8.2%); HPIV (6.2%)	Descriptive and cross-sectional
Sanou [58]	2014–2015	2018	Burkina Faso	<5 years	To study the epidemiology and genetic diversity of viruses and bacteria involved in acute respiratory infections in children under five in Burkina Faso.	(Urban)/584	Hospital	Nasopharyngeal	PCR	HRV (29.8%); HRSV (13%); HAdV (9.8%); HBoV (8.2%); HPIV (7.8%); HMPV (6.2%); HCoV (3.1%); Flu A/B (12.3%); Flu C (2.9%)	Cross-sectional
O’Callaghan-Gordo et al. [59]	2006–2007	2011	Mozambique	<5 years	To present surveillance data on the epidemiology of several respiratory viruses associated with clinical pneumonia in children <5 years admitted to a rural hospital in Mozambique, a malaria-endemic area with high HIV prevalence	(Rural)/394	hospital-based	Nasopharyngeal Aspirate	Multiplex RT-PCR	HRV (41%); HAdV (21%); HRSV (11%); HMPV (8%); Flu A/B (8%); HPIV (7%); EV (4%)	Cross-sectional
Jones et al. [60]	2010–2013	2016	Ghana	<5 years	To concurrently conduct surveillance for severe acute respiratory infection and acute febrile illness (AFI) in three referral hospitals in Ghana to compare influenza-related epidemiologic data from the two syndrome-based surveillance platforms.	(Urban)/1273	Hospital	Naso/Oro-pharyngeal	RT-PCR	Flu A (55%); H1N1 (26%); H3N2 (29%); FluB (45%)	Cross-sectional
Simeon et al. [61]	2017–2018	2021	Namibia	<5 years	To formulate cumulative antibiograms for Intensive Care Units (ICUs) of referral hospitals in Namibia to guide future antibiotic use	(Urban)/976	Hospital	Expectoration	Culture	*K. pneumoniae* (8.8%), *Enterobacter* sp. (22.2%); *P. aeruginosa* (37.5%)	Retrospective Analytical Cross-sectional
Mveang Nzoghe et al. [62]	2020	2021	Gabon	No limit	To Analyze and understand the dynamics of SARS-CoV-2 infection in this unique setting may help other countries in the fight against the COVID-19 pandemic.	Urban/3464	Not indicated	Naso/Oro-pharyngeal and blood	RT-PCR	SARS-CoV-2 (17.2%)	Retrospective
Sebastião et al. [63]	2021	2021	Angola	No limit	To study the prevalence of SARS-CoV-2 in parturients and the risk factors that may be related to SARS-CoV-2 transmission to newborns in Luanda, the capital city of Angola.	Urban/3633	Hospital	Nasopharyngeal	RT-PCR	SARS-CoV-2 (0.4%)	Cross-sectional

**Table 3:** Proportion of pathogens identified in the 43 articles studied, conducted in several countries.

	Proportion (%)	Number of Studies	Number of Countries
Virus			
Human Respiratory Syncytial Virus	0.6–59	28	18
Human rhinovirus	7.5–73	24	14
Influenza virus A/B	0.9–69	19	13
Human adenovirus	0.9–30.8	17	13
Human Parainfluenza virus	2–24	13	10
HCoV (NL63, OC43, 229E, HKU-1)	1.4–13.9	10	8
Human Enterovirus	2.9–25.5	11	7
Human metapneumovirus	1–23.3	11	7
SARS-CoV-2	0.4–44	9	9
Human bocavirus	1.4–16.2	6	5
Bacteria			
*Streptococcus pneumoniae*	1–96	8	9
*Haemophilus influenzae* type b	2.5–54	7	6
*Klebsiella pneumoniae*	1.4–49.9	6	5
*Staphylococcus aureus*	1.7–12.2	2	3
*Pseudomonas aeruginosa*	1.4–37.5	3	3
*Salmonella typhi*	1.6–3	2	1
*Mycobacterium tuberculosis*	0–6.5	1	1
*M. catarrhalis*	0–46.2	1	1
*B. Pertussis*	0–0.1	1	1
*Enterobacter* sp.	22.2	1	1
